# Sustained Experimental Myopia Exacerbates the Effect of Eye Growth on Retinal Ganglion Cell Density and Function

**DOI:** 10.3390/ijms26062824

**Published:** 2025-03-20

**Authors:** Carol Ren Lin, Reynolds Kwame Ablordeppey, Alexandra Benavente-Perez

**Affiliations:** Department of Biological Sciences, SUNY College of Optometry, New York, NY 10036, USA; clin@sunyopt.edu (C.R.L.); rablordeppey@sunyopt.edu (R.K.A.)

**Keywords:** myopia, ganglion cells, marmoset, aging, neurovascular unit

## Abstract

The aim of this study is to describe the effect that sustained myopic eye growth has on the cellular distribution and function of retinal ganglion cells as myopia progresses over time. Ganglion cell density and the photopic negative response (PhNR) were assessed using immunochemistry and electroretinography (ERG), respectively, on twelve common marmoset eyes (*Callithrix jacchus*). Myopia was induced in six eyes using negative defocus (three eyes from 2 to 6 months of age, 6-month-old myopes; three eyes from 2 to 12 months of age, 12-month-old myopes). These six treated eyes were compared to six age-matched control eyes. Marmosets induced with myopia for four months showed a reduced pan-retinal ganglion cell density, which continued to decrease in the peripapillary area of marmosets induced with sustained myopia for ten months. Ganglion cell density decreased as a function of axial length. Full-field ERGs revealed a dampening of the PhNR in the 12-month-old, but not 6-month-old myopes. The myopic changes observed in ganglion cell density and retinal function suggest a reorganization of the ganglion cell template during myopia development and progression that increases over time with sustained myopic eye growth and translates into functional alterations at later stages of myopia development in the absence of degenerative changes. It remains unknown whether these changes positively or negatively impact retinal function and health.

## 1. Introduction

Myopia causes blurred vision at distance and is a pre-eminent risk factor for retinopathies, like glaucoma and choroidal neovascularization, and various maculopathies [1,2], including myopic maculopathy, which is a leading cause of irreversible vision loss. The projected global increase in the prevalence of myopia is predicted to affect over 4.5 billion people by the year 2050 [3,4]. The myopia epidemic has become and will continue to be a significant public health crisis for communities around the world. Despite the significant increase in the prevalence of myopia, the mechanisms underlying the development of myopia and its progression towards retinopathy remain unknown [3], with no early diagnostic markers for preventing myopic pathology [1,2].

The development and progression of myopia leads to both structural and functional ocular alterations. Such myopic alterations include decreased blood oxygen supply [5], inner retina, choroid, and scleral thinning [6,7,8,9,10], decreased microvascular density [5,11,12,13,14], and decreased retinal pigment epithelium density [15,16]. Some of our previous work has described a decrease in astrocyte density along with an increase in glial fibrillary acidic protein (GFAP) spatial coverage associated with thinner retinal nerve fiber layer (RNFL) thickness [17], and decreased peripapillary retinal ganglion cell density [18] in marmosets (*Callithrix jacchus*), a well-established non-human primate model of myopia [19], that were induced with myopia for 4 months and was exacerbated in marmosets induced with myopia for ten months [20]. We have also identified an increase in central and peripheral retinal string vessel density and vascular branchpoint density in eyes induced with myopia for 4 and 10 months [21]. In addition, increased GFAP expression from astrocytes and Müller cells has recently been identified in a mouse model of myopia [22].

Retinal ganglion cells (RGCs) are projection neurons of the vertebrate retina that convey information from other retinal neurons to visual centers in the brain [23,24]. They have a very high metabolism [25] and require the optimal provision of oxygen and nutrients to sustain activity [26]. The special structural architecture of RGCs allows them to be multipotent, with long projecting axons, synaptic terminals to the brain, and elaborate dendritic projection patterns within the retina [27,28]. Under metabolic stress, RGCs are particularly vulnerable to degeneration [29], particularly when coupled with vascular [30,31] and glial dysregulation [32,33,34], which can result in severe retinal neuropathies. This has been demonstrated in both human and experimental models of diseases, including diabetes [35,36,37], glaucoma [26,38,39,40,41], and ocular ischemia [42,43,44,45]. Loss of RGCs within the GCL layer also occurs with age [46,47,48], along with a dendritic reorganization during normal aging [49]. Myopia has been associated with inner and outer retinal thinning [6,50,51,52,53] and increased vascular [16,21,54,55] and glial alterations [17,22,56], which is consistent with other diseases demonstrating similar features occurring parallel to the presence of RGC alterations, dysfunction, or degeneration.

The photopic negative response (PhNR) is the negative wave of the electroretinogram (ERG) immediately following the b-wave, and is indicative of RGC and glial cell function [57] in human [58,59,60] and non-human primate models [61,62]. The PhNR amplitude is used to monitor retinal health in diseases, such as diabetic retinopathy [63], glaucoma [57,64], and retinal ischemic disorders [65], and it corresponds well to inner retinal dysfunction [61]. RGCs contribute the most to the PhNR [59,66,67], making the PhNR a sensitive marker of retinal ganglion cell dysfunction that is highly characteristic of glaucoma [58,61,67,68]. Both myopia and glaucoma are known to cause changes to the inner retinal structure [6,17,50,69,70,71,72,73] and function [8,74,75,76,77]. Full-field ERGs are also known to change with any increase in the axial length of myopic human eyes, especially in eyes with axial lengths greater than 28.00 mm [78]. Axial length is also known to be a greater determinant for full-field ERG responses than refractive error or retinal thickness [78,79].

Our laboratory has reported reduced peripapillary RGC density associated with increasing degrees of myopia and axial lengths in marmosets that were induced with myopia for four months [18] and alterations to inner retinal saturated amplitudes in myopic marmosets that were induced for 4 months compared to controls [80]. However, myopia’s longitudinal effects on RGC density and function, and how they change with increased durations of myopic treatment remains unknown. Here, we aim to describe changes to retinal ganglion cell density and function in marmosets induced with sustained myopia for two periods of time (4 vs. 10 months) to assess the effect of progressive myopic growth on the RGC template, which remains unknown. The results reveal a pan-retinal decrease in retinal ganglion cell density in animals induced with myopia for 4 months that became exacerbated in the peripapillary region of marmosets induced with myopia for 10 months, along with alterations to the photopic negative response (PhNR), both of which are hallmarks of neurodegenerative retinal change.

## 2. Results

**Figure 1:**
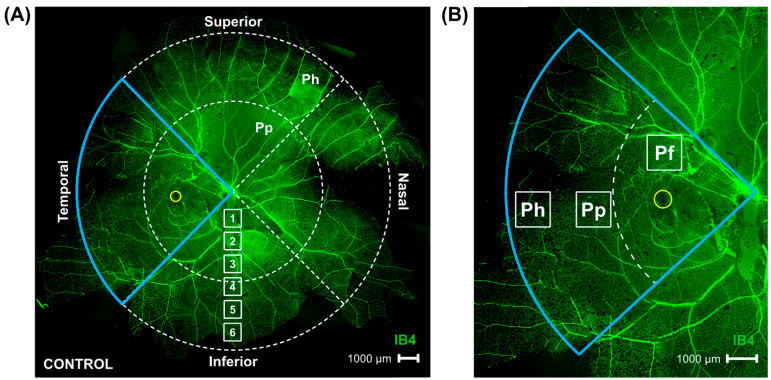
Representative image of whole marmoset retina’s superficial vasculature, demonstrating retinal locations studied. Figure modified from Lin et al. [17]. (**A**) Control marmoset’s superficial vasculature within whole retina, visualized with Isolectin (green; ID: C16 Left eye; scale bar 1000 μm) and consisting of multiple 4× magnification images stitched together using Photoshop. During analysis, the eye was split into four quadrants: Temporal (outlined in blue, also quadrant where the fovea can be found (yellow circle)), Nasal (directly across the optic nerve from temporal quadrant), Superior, and Inferior quadrants. Each quadrant was further split into peripapillary (Pp) and peripheral (Ph) retinal regions. White boxes labeled 1 to 6 show that six focal areas of 40× magnification exist from optic nerve head to periphery. Peripapillary region images were taken at focal area 3 in all four quadrants; peripheral region images were taken at focal area 6 in all four quadrants, and four parafoveal images were taken directly adjacent to the fovea following quadrantal delineation, for total of 12 images taken per retina analyzed. (**B**) Image of temporal retina is shown highlighted in blue, with box 1 (parafovea), box 2 (peripapillary), and box 3 (periphery) showing locations of images gathered in this study.

The age, refractive error, and axial length of all marmoset eyes included in the four treatment groups can be found in Table 1. Magnification correction using a tangential equation was performed to account for the effect that the increased myopic eye size may have on the RGC quantifications. Retinal ganglion cells were located in the ganglion cell layer of both control and myopic marmosets; no displaced ganglion cells were found in other retinal layers. By modeling the expected redistribution of RGCs along the increased surface area, we found a difference of 7% between the predicted number of RGCs (higher) and the actual number of RGCs (lower), which is statistically insignificant and may point to myopic RGC redistribution as the pre-eminent reason for the myopic decline in RGC density noted in this study. A representative image of a complete marmoset retina’s superficial vasculature and areas where the retinas were imaged and quantified can be seen in Figure 1A (modified from Lin et al., 2022 [17]). The location of the parafoveal (Pf), peripapillary (Pp), and peripheral (Ph) retina are shown in Figure 1B.

### 2.1. Parafoveal Ganglion Cell Density Is Lower in Marmosets Induced with Myopia for 4 and 10 Months Compared to Age-Matched Controls

**Figure 2:**
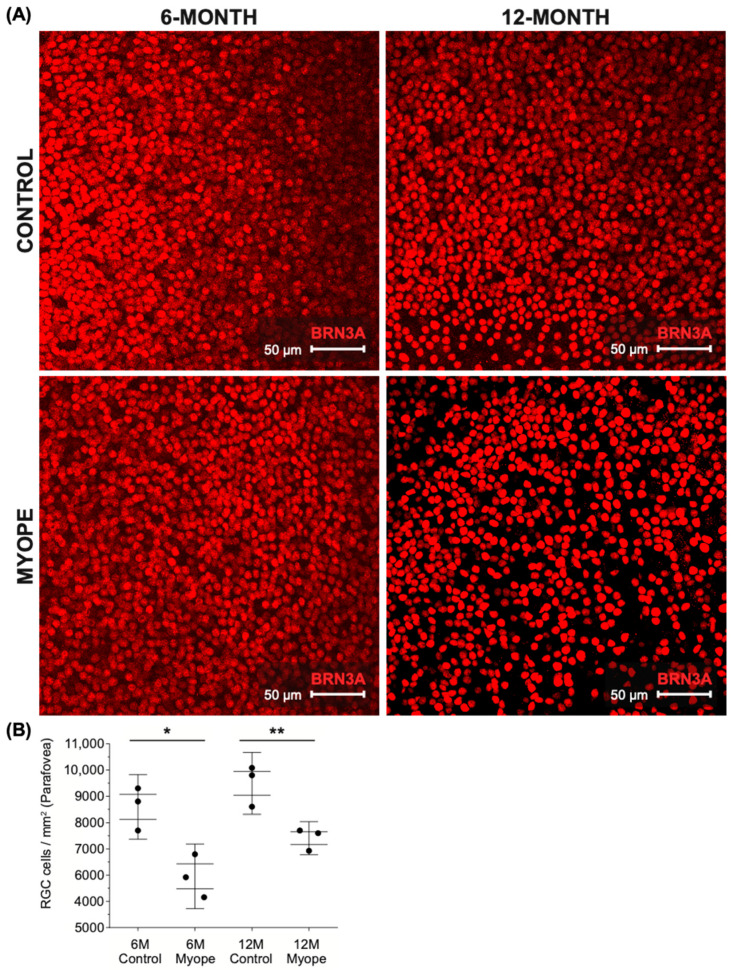
Parafoveal retinal ganglion cell density is significantly decreased in myopic marmosets. * *p* < 0.05, ** *p* < 0.01. Data are shown as box plot with SE as box and SD for whiskers. (**A**) Representative images of parafoveal ganglion cells stained with BRN3A are shown (red; scale bar, 50 μm). Representative image ID tags for control (**top left**: 6-month ID tag Z17 Left, control; **top right**: 12-month ID tag R19 Left) and myopic marmosets (**bottom left**: ID tag E19 Right; **bottom right**: ID tag F20 Left). (**B**) Ganglion cell density is significantly decreased in parafoveal retinas of myopic marmosets with both longer duration of treatment (10 months, *p* < 0.01) and shorter duration of treatment (4 months, *p* < 0.05). There was no significant difference between both groups of myopic marmosets.

Representative images of parafoveal ganglion cells stained with BRN3A can be seen in Figure 2A. The parafoveal ganglion cell density remained unchanged in controls as they grew (6 vs. 12-month-old control eyes, *p* > 0.05) but was significantly lower in the parafoveal retina of myopic marmosets induced with myopia for 4 months (Figure 2B, *p* < 0.05) and remained decreased after 10 months of induced myopia compared to controls (Figure 2B, *p* < 0.01).

### 2.2. Peripapillary Ganglion Cell Density Is Lower in Marmosets Induced with Myopia for 10 Months vs. 4 Months

**Figure 3:**
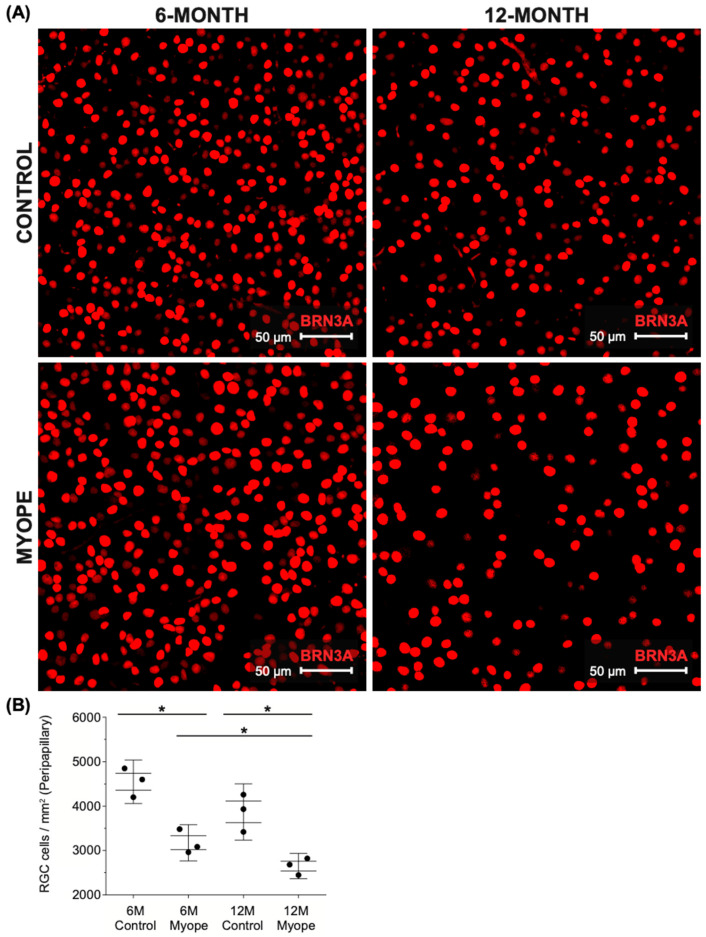
Peripapillary retinal ganglion cell density is significantly decreased in myopic marmosets. * *p* < 0.05. Data are shown as box plot with SE as box and SD for whiskers. (**A**) Representative images of peripapillary ganglion cells stained with BRN3A are shown (red; scale bar, 50 μm). Representative image ID tags for control (**top left**: 6-month ID tag U19 Left, control; **top right**: 12-month ID tag Trike Right) and myopic marmosets (**bottom left**: ID tag E19 Left; **bottom right**: ID tag G20 Left). (**B**) Ganglion cell density is significantly decreased in peripapillary retinas of myopic marmosets with both longer duration of treatment (10 months, *p* < 0.05) and shorter duration of treatment (4 months, *p* < 0.05). There is a significant decrease in older myopic marmoset peripapillary retinal ganglion cell density compared to that of younger myopic marmosets (*p* < 0.05).

Representative images of peripapillary ganglion cells stained with BRN3A are shown in Figure 3A. The peripapillary ganglion cell density remained unchanged in controls as they grew (6 vs. 12-month-old control eyes, *p* > 0.05), but was significantly lower in the peripapillary retina of marmosets induced with myopia for 4 months (Figure 3B, *p* < 0.05). The peripapillary decrease in RGC density was greater in marmosets that experienced sustained myopia for 10 months compared to 4 months (Figure 3B, *p* < 0.05). The myopic peripapillary region was the area that exhibited the greatest percent decrease in RGC density (30–40%) compared to age-matched controls, and compared to the myopic parafoveal (20–30%) and peripheral (20–30%) regions.

### 2.3. Peripheral Ganglion Cell Density Is Lower in Marmosets Induced with Myopia for 4 and 10 Months Compared to Age-Matched Controls

**Figure 4:**
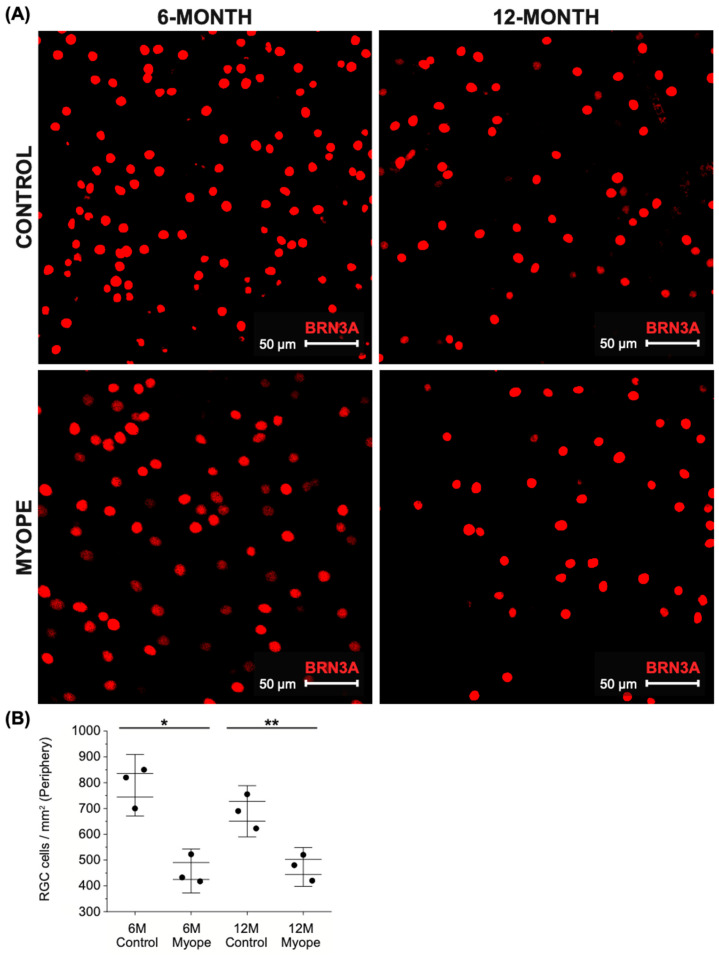
Peripheral retinal ganglion cell density is significantly decreased in myopic marmosets. * *p* < 0.05, ** *p* < 0.01. Data are shown as box plot with SE as box and SD for whiskers. (**A**) Representative images of peripheral ganglion cells stained with BRN3A are shown (red; scale bar, 50 μm). Representative image ID tags for control (**top left**: 6-month ID tag U19 Right, control; **top right**: 12-month ID tag R19 Left) and myopic marmosets (**bottom left**: ID tag E19 Left; **bottom right**: ID tag F20 Left). (**B**) Ganglion cell density is significantly decreased in peripheral retinas of myopic marmosets with both longer duration of treatment (10 months, *p* < 0.01) and shorter duration of treatment (4 months, *p* < 0.05). There is no significant difference between both groups of myopic marmosets (*p* > 0.05).

Representative images of peripheral ganglion cells stained with BRN3A are shown in Figure 4A. Ganglion cell density decreased from the center to the peripheral retina in all treatment groups (Figure 2B, Figure 3B and Figure 4B). The peripapillary ganglion cell density remained unchanged in the controls as they grew (6 vs. 12-month-old control eyes, *p* > 0.05). but was significantly lower in the periphery of myopic marmosets after 4 months (Figure 4B, *p* < 0.05) and 10 months of treatment (Figure 4B, *p* < 0.01). Sustained myopia did not lead to greater changes in peripheral ganglion cell density in myopic animals treated for 4 vs. 10 months (*p* > 0.05).

### 2.4. Dampening of PhNR Amplitude Occurs in Eyes Induced with Myopia for 10 but Not 4 Months

**Figure 5:**
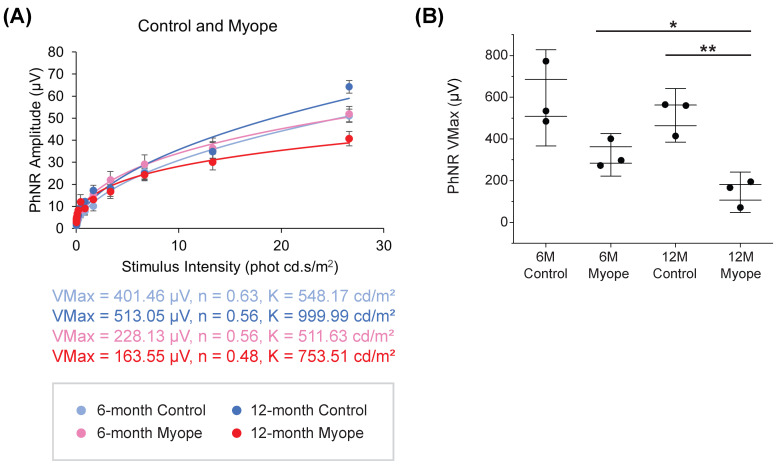
PhNR Vmax amplitudes are significantly decreased in older myopic marmosets treated with negative-powered lenses for 10 months compared to age-matched controls. * *p* < 0.05, ** *p* < 0.01. Light blue signifies 6-month-old control marmosets; pink signifies 6-month-old myopic marmosets treated with negative lens wear for 4 months; dark blue signifies 12-month-old control marmosets; and red signifies 12-month-old myopic marmosets treated with negative lens wear for 10 months. (**A**) Naka–Rushton graph showing PhNR Vmax amplitudes at all stimulus intensities of both myopic cohorts and their age-matched controls (ANOVA *p* < 0.05). (**B**) PhNR Vmax amplitude is significantly decreased in myopic eyes treated for 10 months compared to age-matched controls (*p* < 0.01), but not significantly different in myopic eyes treated for 4 months compared to age-matched controls (*p* > 0.05). PhNR Vmax amplitude is significantly decreased in myopic eyes treated for 10 months compared to myopic eyes treated for 4 months (*p* < 0.05). Data are shown as box plot with SE as box and SD for whiskers.

The PhNR Vmax amplitude differed across all groups in this study, with marmosets induced with myopia for 10 months (red) experiencing the greatest decrease in PhNR amplitude (Figure 5A, *p* < 0.05). The PhNR Vmax amplitude in myopic marmosets induced for 10 months (red) was significantly decreased compared to their age-matched controls (Figure 5B, *p* < 0.01). Myopic eyes induced for 10 months exhibited a significantly decreased PhNR Vmax compared to myopic eyes induced for 4 months (*p* < 0.05). An increased axial length was significantly associated with a decreased PhNR Vmax amplitude in myopic eyes induced for 10 months (R^2^ = 0.98, *p* < 0.05) but not for 4 months (R^2^ = 0.33, *p* = 0.46). The decreased PhNR amplitude in myopic eyes induced for 10 months was also significantly associated with both decreased parafoveal (R^2^ = 0.78, *p* < 0.05) and decreased peripapillary RGC density (R^2^ = 0.71, *p* < 0.05).

## 3. Discussion

This study provides evidence that sustained exposure to negative defocus and myopia development alters ganglion cell density and retinal activity in an established non-human primate model of lens-induced myopia. Compared to age-matched controls, marmoset eyes induced with myopia for four months exhibited a pan-retinal decreased ganglion cell density that became exacerbated in the peripapillary region and was accompanied by a dampened PhNR amplitude in eyes induced with myopia for 10 months.

Ganglion cells are crucial for vision and information transmission [84,85,86,87], especially for central vision in species with foveated retinas, and have been extensively studied in monkeys [88,89,90,91], mice [92,93,94,95,96], cats [97,98,99,100], and humans [46,101,102]. RGCs project their axons along the optic nerve, propagating visual stimuli from the retina to the brain [103]. They are major output cells that process and convey information [104], and comprise subpopulations each with distinct structures and functions [105,106]. RGCs show different levels of contrast sensitivity, visual acuity, and color coding, and a close correlation exists between RGC function and their structural morphology [107,108,109,110,111]. The highest ganglion cell density in primates and humans occurs 0.4–2.0 mm from the foveal center (around 32,000–38,000 cells/mm^2^ in normal young human retinas), and decreases in a horizontally oriented elliptical shape towards the periphery [101]. When the retina’s structural integrity is affected in disease, RGC density and distribution can also be affected and can alter normal vision and physiology. We hypothesize that ganglion cell density, distribution, and function are affected during myopia development and progression.

### 3.1. Pan-Retinal Ganglion Cell Density Decreases in Marmoset Eyes Induced with Myopia for 4 and 10 Months

Retinal ganglion cells can be labeled via a few different markers, including tubulin beta-3 chain (TUBB3) [112], brain-specific homeobox/POU domain protein 3A (BRN3A) [113], and RNA binding protein with multiple splicing (RBPMS) [114]. The Brn3A antibody is a reliable marker to identify RGC nuclei and quantify RGCs in both naïve and damaged retinas [113,115], and is known to label predominantly vision-forming RGCs [116]. Marmoset eyes induced with myopia continuously for 4 or 10 months exhibited a similar decrease in BRN3A+ retinal ganglion cell density compared to age-matched controls, which remained significant after correcting for the effect of myopic magnification. Lower RGC cell counts along with thinner inner retinal thicknesses and decreased visual field sensitivity have been observed in macaques with induced glaucoma [73]. However, specific information describing myopic RGC numbers and function remains to be determined, making direct measurements of RGCs and RGC density in experimental myopia crucial to understanding the sequence of events taking place prior to the development of myopic retinopathy, as well as the etiology of myopic visual decline.

The eyes of untreated controls aged 6 and 12 months exhibited similar ganglion cell densities across the retina and the densities aligned with those reported in older marmosets [117], as well as with studies from our laboratory [18]. RGC density in animals induced with experimental myopia, however, decreased by approximately 30% in all retinal areas studied (parafovea, peripapillary, and periphery). Eyes induced with myopia for 10 months experienced greater decreases in RGC density in the peripapillary region, compared to myopic eyes induced with myopia for 4 months. The peripapillary region of myopic eyes treated for 4 and 10 months with negative lens wear experienced exacerbated decreases in RGC density. We hypothesize that this decrease in RGC density may be due to ganglion cell redistribution from extrinsic factors, such as increased myopic growth and mechanical stress. Susceptible areas for the effects of ocular mechanical stress include the lamina cribrosa and the peripapillary sclera [118,119,120], which are both known to be altered in myopia progression [52,121,122] and are directly adjacent to the peripapillary retina measured in the marmosets of this study. This is of particular importance because the peripapillary retina is the main region where the decrease in RGC density was greater in the study marmosets treated for 10 months versus those treated for 4 months. The peripheral RGC densities did not worsen with prolonged myopia despite axial elongation. This could be due to the majority of retinal ganglion cells being located in the posterior pole [70], leading to peripapillary RGCs being the most affected with sustained myopia.

Myopic growth is known to be non-uniform, via posterior/axial expansion [123,124] and sagittal enlargement in the equatorial region [15]. While the specific locations where the retinas in this study experience greater elongation and/or expansion are unknown, larger eyes normally contain a greater retinal surface area subtending a given visuospatial field [125]. Myopic stretch could cause the same number of RGCs to have spread over a larger surface area, resulting in less densely packed ganglion cells [69] (observed in this study) or larger cell bodies (not observed in this study). The myopic marmoset retinas in our study exhibited a 10% increase in surface area compared to age-matched controls. In the literature, chicks induced with form-deprivation myopia exhibit reduced RGC cell density (greater in the peripheral versus the mid-peripheral retina) compared to control eyes [126], sampled every 2.5 mm along the horizontal and 0.6 mm along the vertical meridians of whole retinal flat mounts. Additionally, these myopic chick eyes exhibited greater RGC dendritic arborization [126]. This decreased ganglion cell density with compensatory increased arborization could explain why chick myopic eyes have been described to have poorer visual function, though this was not studied in the present work. Decreased ganglion cell density and thickness have also been identified in human myopic eyes [125], although the density calculations were not performed using histology but ganglion cell layer tissue volume. A histological study of human eyes found that within the central and peripheral nervous system, a relationship exists between cell numbers, size, and density [127,128], meaning the larger the organ, the larger the soma size of the cells within the organ, and the less densely packed the cells. As presented, myopia appears to manifest with decreased ganglion cell densities across species, and the implications of decreased RGC density on long-term healthy vision need more investigation in the future.

The hallmark disease exhibiting a decrease in ganglion cell counts is glaucoma, which causes RGCs to become damaged and undergo cell apoptosis leading to death at severe stages [129,130,131,132,133]. RGCs are inherently mechanosensitive cells [134,135,136], and any extended duration of biomechanical stressors (like myopic eye growth) can potentiate the development and progression of RGC dysfunction.

A possible confounding variable in the RGC density changes noted could be interanimal variability. However, the greater drop in peripapillary RGC density observed in marmosets induced with myopia for 10 months vs. 4 months might also be due to the known effect that aging has on retinal ganglion cell function, in particular the age-related losses of neurons in the inner retina [137]. This may also explain why age is a significant risk factor for the development of vision defects. With age, the risk of both pathological and non-pathological RGC loss occurrence increases [137,138]. The locational patterns and the extent of age-related neuronal loss are known to be different in control and glaucomatous mice retinas, with control eyes showing diffuse RGC loss occurring at a greater intensity after 12 months of age and glaucomatous eyes showing patchy, non-quadrant-preferential RGC loss occurring steadily over time and slowing down after 15 months of age [139]. Another study of mouse RGCs showed decreasing RGC dendritic arborization and a minimal decline in RGC numbers with age [140]. Not all mammals experience the same effects of aging on RGC density. Human studies have shown an age-related histological loss of RGCs with no preference for the size or type of RGC [46,137], which presents with concurrent thinning of retinal nerve fiber layers [50,137]. Findings from rhesus monkeys [141] and quoka wallabys [142] have shown no change in RGC numbers with age. The first changes from aging that occur in a monkey brain are visual cortex myelin and glial cell degeneration [143,144,145,146], followed by a decrease in visual cortex ganglion cell density. This is similar to the sequence of events seen with physiological aging in the retina, beginning with retinal glial changes during early aging [147,148], and followed by retinal ganglion cell structural abnormalities and functional deficits with time [46,149]. However, it is important to highlight that there was no age-related ganglion cell loss seen in our control marmoset eyes. Greater periods of study may be necessary to understand the effects of age on ganglion cells in marmoset retinas.

### 3.2. Photopic Negative Response (PhNR) Amplitude Is Significantly Dampened in Marmosets Induced with Myopia for 10 Months, but Not in Marmosets Treated for 4 Months

Electroretinography (ERG) is crucial for evaluating retinal function and assessing the electrical potentials from retinal cells to light stimulation [63]. The Photopic Negative Response (PhNR) is the negative potential following the b-wave that originates from retinal ganglion cells (RGCs) and glial cells, corresponding to inner retinal function. The PhNR in particular is useful for detecting early glaucoma and evaluating the retinal function of patients with retinal ischemic disorders [58,60,61,68,150], and corresponds with the amount of inner retina dysfunction [61].

Myopic marmosets treated for 10 months exhibited a dampening of the PhNR amplitude compared to their age-matched controls. These changes were not observed in marmosets induced with myopia for 4 months. The PhNR amplitude is the negative response of the photopic electroretinogram (ERG), and is indicative of inner retinal cone-related RGC and glial cell function [57]. Amacrine cells have also been shown to contribute to the PhNR amplitude by modulating information between RGCs and bipolar cells [61]. The full-field PhNR amplitude is also associated with the mean deviation of visual fields and retinal nerve fiber layer thickness [151]. In human studies [57,64] of sustained IOP elevations and subsequent reductions in IOP, the PhNR amplitude decreases then increases, with residual slower implicit times that are likely due to these processes being glial cell-mediated [61]. Improved glial function is proposed to be one mechanism by which PhNR amplitude shows improvements following IOP reductions, possibly via more effective ion homeostatic control; however, the major component of the PhNR is likely from RGCs [57]. The P1 and N1 amplitudes (the two components of the PhNR) have been shown to be lower in human myopic eyes [78,152], with no alteration in implicit times [153,154]. Full-field ERGs are known to change with any increase in the axial length of myopic human eyes, especially in eyes with axial lengths greater than 28.00 mm [78]. Axial length is also known to be a greater determinant for full-field ERG responses than either refractive error or retinal thickness [78,79]. To our knowledge, our laboratory is among the first to specifically study the PhNR in an experimental model of sustained myopia; most studies to date have only provided evidence of reduced electroretinogram function, with no specific mention of PhNR amplitudes. A recent study from our laboratory investigating the ERG function of marmosets over a range of ages has shown alterations to inner retinal saturated amplitudes in myopic marmosets compared to controls [80], similar to findings in this study’s marmosets.

A decreased PhNR amplitude is associated with both decreased parafoveal and peripapillary RGC densities, as well as increasing axial lengths, in myopic marmosets treated for 10 months. The axial elongation of myopic eyes can cause inner retinal stretch and subsequently, a reduced sampling density of retinal neurons and their functional physiology [155]. Myopic eyes experience asymmetric stretch, with both peripheral and macular pathologies often occurring in degenerative myopia [13]. We postulate that sustained myopic retinal stretch, known to cause cellular redistribution and morphological changes, is likely to cause functional alterations as seen in the decrease in PhNR amplitudes seen only in myopic marmoset eyes that were treated with negative lens wear for 10 months (but not exhibited in myopic eyes that were treated for 4 months). Myopic marmosets treated for four months may not have experienced a long enough exposure to negative defocus to warrant significant associations between RGC density and axial length and retinal physiology in the peripheral region, but have been shown to experience a non-significant trend towards PhNR dampening [80,156].

The PhNR amplitude alteration noted with electroretinography in the older myopic marmosets points to possible functional alterations of the photoreceptors, glial cells, and ganglion cells occurring in the absence of stark myopic pathology. Understanding the longitudinal effect of progressive experimental myopia on retinal cellular structures in tandem with retinal circuitry is critical for managing the conversion from high to pathological myopia. Considering ocular axial length may also serve to be useful in predicting the severity of the functional alterations in retinal ganglion cells in early myopic degeneration.

## 4. Materials and Methods

### 4.1. Marmoset Model of Myopia

Twelve marmoset eyes were studied and randomly grouped into treated or age-matched untreated controls: three 6-month-old myopic eyes (6 m Myope) were induced with myopia for 4 months (from 2 to 6 months of age), three age-matched 6-month-old untreated controls (6 m Control), three 12-month-old myopes induced with myopia for 10 months (12 m Myope) from 2 to 12 months of age, and three age-matched 12-month-old controls (12 m Control) (Table 1). Animals were induced with myopia using full-field negative single-vision soft contact lenses (either −5D or −10D) [17]. Statistical power analysis using published data from our laboratory indicates that 3 animals per experimental group provide 80% power for our statistical analysis (*n* = 3 younger control, *n* = 3 younger myope, *n* = 3 older control, *n* = 3 older myope) [17]. There were no exclusions of animals, experimental units, or data points reported. All animal care, treatment, and experimental protocols were approved by the SUNY College of Optometry Institutional Animal Care and Use Committee (IACUC), and performed as recommended by the ARVO Statement for the Use of Animals in Ophthalmic and Vision Research, the US National Research Council’s Guide for the Care and Use of Laboratory Animals, the US Public Health Service’s Policy on Humane Care and Use of Laboratory Animals, and the Guide for the Care and Use of Laboratory animals.

At baseline and end of treatment, cycloplegic refractive error (Rx) and ocular axial length (AL) were measured using the Nidek ARK-900 autorefractor (Nidek Co., LTD, Gamagori, Japan) and an ultrasound biometer (A-scan ultrasound 25 MHz, Panametrics, NDT Ltd., Waltham, MA, USA) respectively, prior to tissue collection under anesthesia (alphaxalone, 15 mg/kg, intramuscular, Zoetis, Parsippany, NJ, USA).

### 4.2. Electroretinograms (ERGs)

ERG measurements were performed on all marmosets at end of treatment. Marmoset ERGs were recorded using the Espion electrodiagnostic system (Diagnosys LLC, Lowell, MA, USA), consisting of the Espion Ganzfeld ColorDome and its accompanying computer program. The photopic ERG protocol used in this study was the brief 75 (13 steps, 0.00625–26.624 (cd*s)/m^2^) consisting of increasing flash intensities over 13 steps. The stimulus was a 4-millisecond-long white stimulus presented on a constant white background.

Marmosets were dilated with cyclopentolate hydrochloride (Alcon, Inc. Fort Worth, TX, USA) 1 h prior to measurements, and injected with glycopyrrolate (0.01 mg/kg, IM) and acepromazine (2.5 mg/kg, IM) 25 min prior to ketamine injection. Ketamine (50 mg/kg, IM) was subsequently administered and placed on a warm heating pad after confirming adequate and safe anesthesia, with vitals monitored every 15 min throughout ERG recordings duration. For protecting corneal health, 1% proparacaine hydrochloride (Akorn Inc., Lake Forest, IL, USA) was instilled in both eyes, gold wire electrodes were placed on both corneas, and a black light-proof cloth was placed over the reference eye. A grounding electrode was inserted subcutaneously into the back, and ERG recordings were gathered. At the conclusion of the ERG recording, topical erythromycin ophthalmic ointment was applied to marmoset eyes and marmosets were monitored until full recovery from anesthesia.

The Photopic Negative Response (PhNR) amplitudes were measured at all steps. The PhNR reflects the functioning of ganglion and glial cell responses, and is defined as the negative trough located between the b-wave and d-wave, usually found at around 65 milliseconds. Implicit times for all flash intensities and stimulus parameters were recorded, and response data was fit to a Naka–Rushton equation (below) for plotting intensity–response data [157], as successfully used in previous marmoset experiments in our laboratory [80,156]:

(1) $$V\left(I\right)=\frac{V_{max}I^{n}}{I^{n}+K^{n}}$$

Variables: *I* stands for the stimulus intensity, *V* stands for the amplitude at intensity *I*, Vmax stands for the saturated amplitude, *K* stands for the semi-saturation constant (stimulus intensity at which half of the saturated amplitude is reached), and n stands for the slope of the function.

### 4.3. Tissue Collection

At the end of treatment, eyes were enucleated and placed in phosphate-buffered saline (PBS; ThermoFisher, Waltham, MA, USA). Dissected retinas were fixed in Paraformaldehyde (PFA) 4% (Santa Cruz Biotechnology, Dallas, TX, USA) in PBS for 30 min, washed five times for 30 min each with PBS, and incubated with 5% normal donkey serum (Sigma Aldrich, St. Louis, MO, USA), PBS, and 0.5% Triton X (Sigma Aldrich, St. Louis, MO, USA) blocking buffer for one hour to avoid non-specific antibody binding. Following blocking, retinal tissue was incubated with primary antibodies diluted in blocking buffer at 4 °C for 4 days. The antibody used in this study was goat Brain-Specific Homeobox/POU Domain Protein 3A (BRN3A) (1:500, Catalog #sc-39184, Santa Cruz Biotechnology, Dallas, TX, USA) to stain all marmoset retinas. After the primary antibody incubation period, the retinas were washed six times for 10 min each with PBS and incubated with donkey-anti goat secondary antibody conjugated with Alexa 594 (1:200, ThermoFisher, Catalog #A11058, Waltham, MA, USA) for 2 days. Retinal tissues were then washed one time for 30 min, and 6 times for 10 min each with PBS. Retinas were inspected for signs of debris, and consistent tissue thickness was achieved by removing vitreous prior to plating onto SuperFrost slides (ThermoFisher, Waltham, MA, USA). Cover slips were then placed on objectives with DAPI mounting medium (SKU: H-1200-10, Vector Laboratories, Newark, CA, USA), permitted to self-seal, and stored at −20 °C.

### 4.4. Confocal Microscopy and Image Acquisition

Immunohistochemical samples were imaged using Olympus FV1200 MPE confocal microscope (Olympus Corporation, Tokyo, Japan). The images were gathered, and analyses were performed in a randomized order by one blind investigator (CL). After enucleation, right and left eyes were kept separately, and denotation of the temporal region was marked by the presence of the foveal pit. Twelve images (317 μm × 317 μm along the horizontal plane, and 20 μm along the vertical plane) were taken from each of the twelve retinas imaged. Multiplane z-series were collected using the 40× objective, with each section spaced 1 μm apart. These 20 sections were processed by the confocal microscope to form single z-stacks of images subtending the desired portion of the specimen. Images were processed using Fiji software version 2.9.0. Ganglion cell density (RGC nuclei/mm^2^) was assessed by imaging all four retinal quadrants (temporal, nasal, superior, and inferior) in the periphery, peripapillary, and parafoveal retina. Temporal region was determined based on the location of the fovea. Nasal region is directly opposite of the temporal region, and depending on the eye, superior and inferior retina were categorized. This regional analysis was performed with the goal of identifying local changes that might occur in myopic eyes due to their asymmetric eye growth pattern.

### 4.5. Image and Statistical Analysis

Retinal ganglion cell density was calculated as the number of ganglion cell nuclei in every image frame using the Fiji cell counter function and converted to ganglion cells/mm^2^. A correction for magnification along the two-dimensional plane was performed to account for myopic retinal growth using a tangential equation. Data were assessed for normality and analyzed using student *t*-test and one-way analysis of variance (ANOVA), and post-hoc analysis with Tukey tests at the level of α = 0.05 was used to examine the differences between treatment and control groups. Pearson’s linear correlation was used to explore the relationship between effective age, axial length, refractive error, and astrocyte measurements. Figures were made using OriginPro 2024b software (OriginLab, Northampton, MA, USA) and were assembled in Adobe InDesign (Adobe, San Jose, CA, USA). An algebraic analysis was also performed to account for increased surface area from myopia, assuming that non-myopic marmosets have spherically shaped eyes and that myopic marmosets have more ellipsoid eyes.

## 5. Conclusions

Taken together, the pan-retinal decreased ganglion cell density and dampening of the PhNR amplitude observed in myopic marmosets experiencing sustained myopic growth for 4 months suggest that both structural and functional changes are taking place in the ganglion cell template in the absence of myopic pathology. Myopic marmosets treated for 10 months with negative lens wear experienced exacerbated decreased peripapillary ganglion cell density and similarly decreased parafoveal and peripheral ganglion cell density compared to myopic eyes treated for 4 months with lens wear, as well as to their age-matched controls. We hypothesize that a redistribution of the ganglion cell template is occurring during myopia development and progression in this non-human primate model, leading to lower ganglion cell density and PhNR amplitudes, which in turn may represent early signs of anatomical and physiological changes in myopic eyes. The amount of harm incurred by these changes, and whether or not function continues to diminish with myopia progression remains to be seen. Future studies will aim to evaluate quantitatively the changes that occur with older, more myopic eyes.

## Figures and Tables

**Table 1:** Myopic marmosets initiated lens wear at 10 weeks old (72.0 ± 5.5 days) following an established protocol [81,82]. Daily morning contact lens insertion occurred between 8 and 10 am. The lights (700 lux) were turned on at 10 am after the lenses were inserted, and were subsequently removed 9 h later at lights-off each day (9 h of light/15 h of dark). The contact lenses were either a 3.6 or 3.8 mm base curve and a 6.5 mm diameter, made of methafilcon A (55% water content, Dk: 17), and fit 0.10 mm flatter than the flattest keratometry measurement. No corneal complications were observed in any of our treated myopic animals in this or earlier marmoset studies [81,82,83].

6 m Control ID, Eye	Eye Length (mm)	Refraction (D)	Gender	Age (Days)	6 m Myope ID, Eye	Eye Length (mm)	Refraction (D)	Gender	Age (Days)
U19, Right	9.83	−1.73	Male	249	U17, Right	10.24	−3.59	Male	220
U19, Left	9.79	−2.24	Male	249	E19, Right	10.86	−4.29	Male	221
Z17, Left	9.81	−0.41	Male	214	E19, Left	10.80	−3.64	Male	221
AVG ± SD	9.81 ± 0.0	−1.46 ± 0.9		237.33 ± 20.2	AVG ± SD	10.63 ± 0.3	−3.84 ± 0.4		220.67 ± 0.6
						*p* < 0.05	*p* < 0.05		*p* > 0.05
**12 m Control ID, Eye**	**Eye Length (mm)**	**Refraction (D)**	**Gender**	**Age (Days)**	**12 m Myope ID, Eye**	**Eye Length (mm)**	**Refraction (D)**	**Gender**	**Age (Days)**
Trike, Right	10.22	−1.12	Female	382	F20, Left	11.12	−7.86	Male	396
R19, Left	10.22	−1.04	Male	381	G20, Left	11.33	−5.715	Female	396
P16, Right	11.21	+1.22	Female	396	H20, Left	11.05	−11.70	Female	420
AVG ± SD	10.56 ± 0.5	−0.31 ± 1.3		386.33 ± 8.4	AVG ± SD	11.17 ± 0.1	−8.42 ± 3.0		404 ± 13.9
						*p* < 0.001	*p* < 0.001		*p* > 0.05

## Data Availability

The original contributions presented in this study are included in the article. Further inquiries can be directed to the corresponding author.
